# Body height estimation from automated length measurements on standing long leg radiographs using artificial intelligence

**DOI:** 10.1038/s41598-023-34670-2

**Published:** 2023-05-25

**Authors:** Sebastian Simon, Barbara Fischer, Alexandra Rinner, Allan Hummer, Bernhard J. H. Frank, Jennyfer A. Mitterer, Stephanie Huber, Alexander Aichmair, Gilbert M. Schwarz, Jochen G. Hofstaetter

**Affiliations:** 1 Michael Ogon Laboratory for Orthopaedic Research, Orthopaedic Hospital Vienna-Speising, Speisinger Straße 109, 1130 Vienna, Austria; 2 2nd Department, Orthopaedic Hospital Vienna-Speising, Speisinger Straße 109, 1130 Vienna, Austria; 3 Unit for Theoretical Biology, Department of Evolutionary Biology, University of Vienna, Djerassiplatz 1, 1030 Vienna, Austria; 4 ImageBiopsy Lab GmbH, Zehetnergasse 6/2/2, 1140 Vienna, Austria; 5 Center for Anatomy and Cell Biology, Medical University of Vienna, Währingerstraße 13, 1090 Vienna, Austria

**Keywords:** Bone, Translational research, Anthropology, Radiography

## Abstract

Artificial-intelligence (AI) allows large-scale analyses of long-leg-radiographs (LLRs). We used this technology to derive an update for the classical regression formulae by Trotter and Gleser, which are frequently used to infer stature based on long-bone measurements. We analyzed calibrated, standing LLRs from 4200 participants taken between 2015 and 2020. Automated landmark placement was conducted using the AI-algorithm LAMA™ and the measurements were used to determine femoral, tibial and total leg-length. Linear regression equations were subsequently derived for stature estimation. The estimated regression equations have a shallower slope and larger intercept in males and females (Femur-male: slope = 2.08, intercept = 77.49; Femur-female: slope = 1.9, intercept = 79.81) compared to the formulae previously derived by Trotter and Gleser 1952 (Femur-male: slope = 2.38, intercept = 61.41; Femur-female: slope = 2.47, intercept = 54.13) and Trotter and Gleser 1958 (Femur-male: slope = 2.32, intercept = 65.53). All long-bone measurements showed a high correlation (*r* ≥ 0.76) with stature. The linear equations we derived tended to overestimate stature in short persons and underestimate stature in tall persons. The differences in slopes and intercepts from those published by Trotter and Gleser (1952, 1958) may result from an ongoing secular increase in stature. Our study illustrates that AI-algorithms are a promising new tool enabling large-scale measurements.

## Introduction

The estimation of human stature from long bone length measurements is a common task in forensics or biological anthropology, and it can also be used to assess body mass index for hospitalized and bedridden patients. Because of variation in body form between human populations, it is essential to base the inference of stature on formulae derived for the population of interest. Indeed, several authors have demonstrated that estimation is affected by differences between human populations^1–3^. Different regression formulae are available in the literature for stature estimation for a number of human populations, based on data measured either from autopsied corpses^4,5^ or from skeletal remains^1,6^. However, existing studies are mostly based on rather small sample sizes and mostly, data is collected from deceased bodies; very few studies used data from living humans^7^.

Adoption of algorithms based on Artificial Intelligence (AI) has become increasingly widespread in various fields of medical and biological research. The benefit of these new methods lies in handling large amounts of data in a fully automated way. Accurate measurement of bone length is traditionally done manually from standing long leg radiographs (LLRs) for living humans or from skeletal material as well as from cadavers for deceased persons. This manual measurement process is both time-consuming and sometimes poorly reproducible because of the use of different software applications and different measurement techniques^8,9^. Manual landmark placement may also lead to high inter-observer variability. A recently published AI-based algorithm automatizes length and angle measurements on LLRs, which enables using much larger datasets and produces standardized outputs^10–12^. In general, the application of AI technology in medicine facilitates the analysis of large imaging datasets such as radiographs, computer-tomography (CTs) or Magnetic Resonance Images (MRIs) in medicine. However, to date, no study has been published on the use of AI technology for height estimation based on radiographic measurements in a large human sample.

The frequently used regression formulae derived by Trotter and Gleser in 1952 and 1958 are considered to be suitable for persons of European ancestry^13–15^ and are applied to estimate stature from skeletal remains. Trotter and Gleser suggested not to estimate stature by determining the average of estimates obtained from several equations, each of which is based on a different bone or on a combination of bones^1^. Although the formulae derived by Trotter and Gleser^1,15^ are considered to be quite reliable, these regressions were derived in the more than half a century ago, and average human stature has increased markedly since then, especially in high-income countries^16,17^. It is therefore a reasonable suggestion that these formulae may need to be adapted due to the secular change in stature^18^.

In this study, we aimed to derive updated regression formulae to infer stature for humans of European ancestry based on long bone measurements from living patients. For this purpose, we used measurements from LLRs taken between 2015 and 2020. We based the regressions on a large sample of more than 4000 adults and applied an AI-based algorithm to acquire tibial length, femoral length and total leg length for this patient sample. We then compared these newly derived regression formulae to existing ones in the literature.

## Results

### Patient demographics

**Figure 1:**
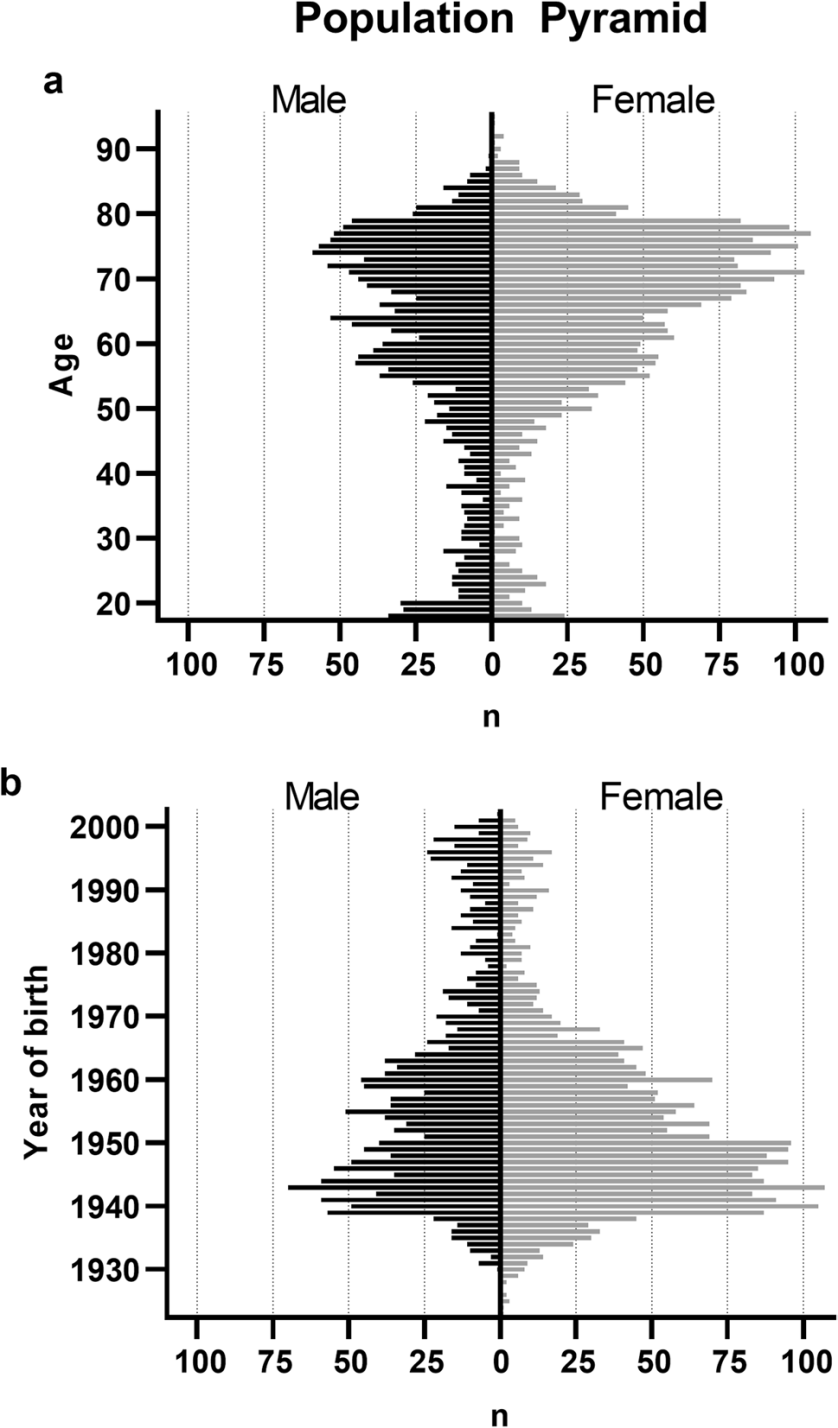
Distributions of (**a**) age at the time of image acquisition and (**b**) year of birth for the female and male patient samples.

**Figure 2:**
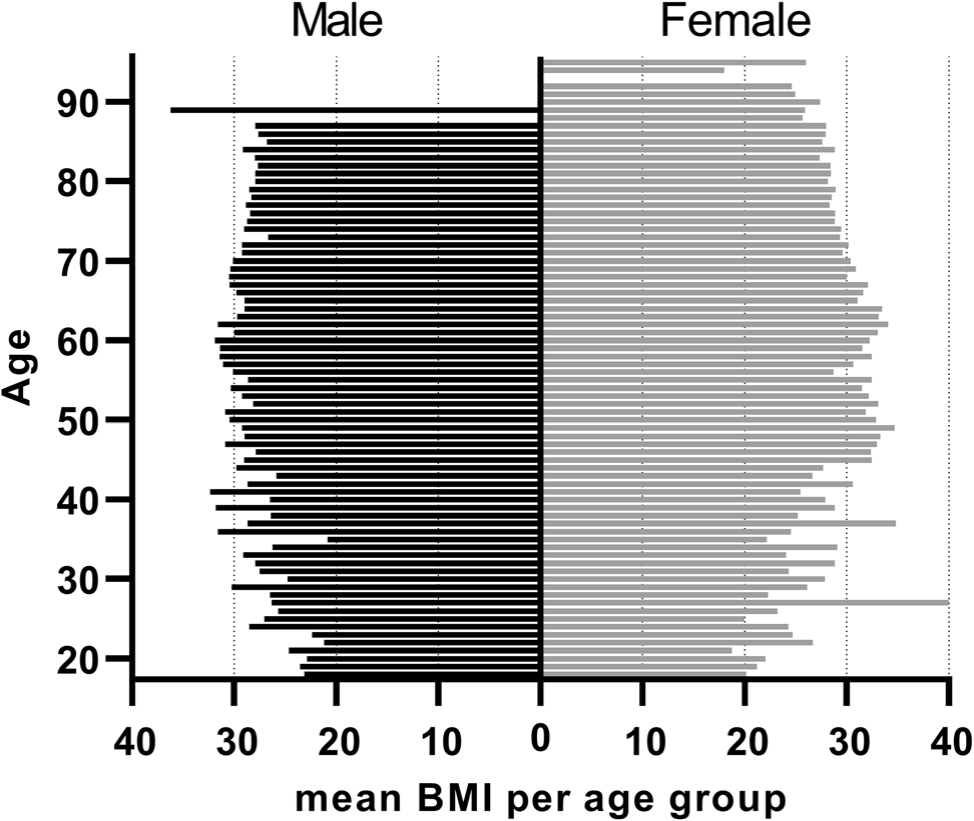
Distribution of mean BMI per age group, separately for the male and female patient samples.

**Figure 3:**
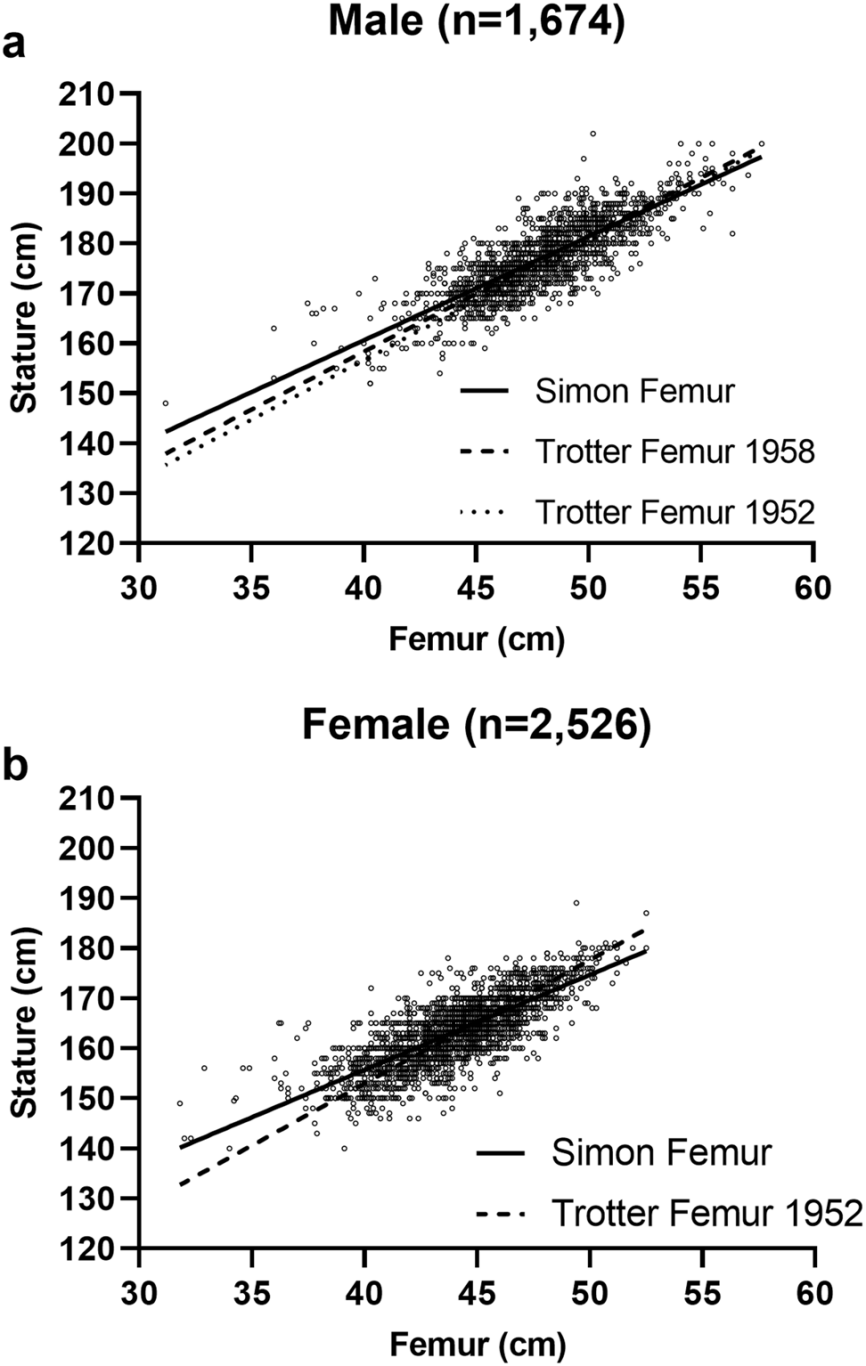
Scatterplot of femur and stature measurements for the male (**a**) and female (**b**) sample and the estimated regression lines based on these data. For comparison, the regression lines of the current study (Femur Simon) are shown together with regression lines from the literature [Trotter and Gleser Femur for females (1952) and males (1958), Trotter and Gleser Femur only for males (1952)].

Of the 4200 LLRs included in the final analysis, 2526 (60.1%) were from female patients and 1674 (39.9%) were from male patients. All included patients were between 18 and 95 years old and born between 1923 and 2002 with a median age of 66 years (Fig. 1). The mean BMI was 29.44 kg/m^2^ (± 5.8 kg/m^2^ SD) and the mean height was 168.9 cm (± 9.6 cm SD). Summary statistics of patient demographic variables and total numbers of left, right and bilateral radiographs, which were used in this study, are presented in Table 1. Mean BMI was similar across age groups (Fig. 2). Scatterplots for femur length and stature, separately for males and females, are shown in Fig. 3.

**Table 1:** Summary statistics for the demographic variables.

Variable	Male (n = 1674)	Female (*n* = 2526)
Age (years)	64.00 (18; 89)	68.00 (18; 95)
BMI (kg/m^2^)	28.74 (± 5.09)	29.92 (± 3.14)
Stature (cm)	177.14 (± 7.40)	163.41 (± 6.50)
Left leg (n)	680	1093
Right leg (n)	716	1223
Bilateral (n)	278	210
Femur length (SD)	47.07 (± 4.31)	43.06 (± 4.31)
Tibia length (SD)	37.78 (± 3.63)	35.02 (± 2.78)
Leg length (SD)	84.66 (± 7.75)	78.59 (± 5.89)

Indicated are the median for age (minimum; maximum) and the mean ± standard deviation (SD) for body mass index (BMI) and stature, separately for males and females. Indicated are also the numbers of left, right and bilateral radiographs used in this study. The data set consisted of 1674 male and 2526 female radiographs in total.

### Regression results

The linear regression equations for the estimation of stature in our sample based on either one or two bone lengths are presented in Table 2.

**Table 2:** Stature estimation equations based on femur, tibia, leg length and femur + tibia length, respectively.

Variable	Male equation	Female equation
Femur	2.078*Femur + 77.49	1.897*Femur + 79.81
Tibia	2.415*Tibia + 86.35	2.301*Tibia + 84.16
Leg length	1.201*Leg length + 73.92	1.112*Leg length + 75.80
Femur + tibia	1.212*(Femur + Tibia) + 73.48	1.121*(Femur + Tibia) + 75.45

Correlations between stature and long bone lengths were consistently larger than 0.7 for all considered long bones in males and females. For the male sample, this correlation was *r* = 0.82 (95% CI 0.80–0.83) for the femur, *r* = 0.80 (0.79–0.82) for the tibia, *r* = 0.84 (0.82–0.86) for total leg length and *r* = 0.84 (0.83–0.86) for tibia + femur. The slopes and intercepts are the averages of left and right. The intercept for the femoral regression was 77.49 in our equation for males, compared to an intercept of 65.53 in Trotter and Gleser (1958), and an intercept of 61.41 in Trotter and Gleser (1952). The slope was 2.08 in our equation for males, compared to a slope of 2.32 in Trotter and Gleser (1958) and a slope of 2.38 in Trotter and Gleser (1952), see Fig. 3a.

The correlations between long bone length and stature for the female sample were *r* = 0.77 (95% CI 0.76–0.79) for the femur, *r* = 0.76 (0.75–0.78) for the tibia, *r* = 0.80 (0.78–0.81) for leg length and *r* = 0.80 (0.78–0.81) for tibia + femur. The intercept for the femoral regression in females was 79.81 in our sample, compared to an intercept of 54.13 in Trotter and Gleser (1952). The slope of the femoral regression for females was 1.90 in our equation, compared to a slope of 2.47 in Trotter and Gleser (1952), see Fig. 3b.

Average stature of the male subsample was 177 cm (± 7.4 cm SD), with a range from 148 to 202 cm. For females, average stature was 163 cm (± 6.5 cm SD), ranging from 140 to 189 cm. Detailed tables summarizing the stature distributions as well as the corresponding averages of the femur, tibia, leg and tibia + femur measurements for the male (Table 3) and female (Table 4) samples are included below.

**Table 3:** Distribution of stature and means of long bone measurements for male (femur, tibia + femur, leg length, tibia length) for each stature level (each cm).

Stature	*n*	Male (n = 1674)			
		Femur	Tibia + Femur	Leg length	Tibia
148	1	31.20	68.00	68.30	36.80
152	2	40.30	70.60	71.05	30.30
153	1	36.00	68.10	68.40	32.10
154	1	43.40	76.00	76.50	32.60
155	4	40.20	72.30	72.63	32.10
156	2	39.95	71.40	71.70	31.45
157	2	41.85	75.40	76.05	33.55
158	3	41.57	74.10	74.17	32.53
159	4	42.00	75.28	75.48	33.28
160	14	42.60	76.31	76.62	33.71
161	2	43.25	77.45	77.60	34.20
162	2	44.55	78.70	79.15	34.15
163	7	42.60	76.16	76.53	33.56
164	3	44.77	78.23	78.47	33.47
165	36	44.09	78.57	79.03	34.48
166	16	43.83	79.33	79.81	35.50
167	32	44.77	79.76	80.08	34.99
168	64	45.10	79.86	80.26	34.76
169	23	45.64	80.92	81.38	35.28
170	108	45.71	81.40	81.83	35.69
171	45	46.24	82.27	82.66	36.03
172	104	46.17	82.36	82.76	36.19
173	62	47.04	83.44	83.89	36.40
174	65	46.93	83.66	84.06	36.74
175	96	47.03	83.95	84.42	36.91
176	112	47.63	84.80	85.22	37.17
177	47	48.08	85.61	86.00	37.53
178	113	48.21	85.85	86.29	37.64
179	45	48.44	86.37	86.80	37.94
180	148	48.82	87.12	87.52	38.30
181	37	49.35	88.07	88.51	38.73
182	58	49.85	89.08	89.56	39.23
183	82	49.94	89.11	89.49	39.17
184	46	50.39	90.02	90.46	39.63
185	72	50.06	89.75	90.23	39.69
186	54	50.88	91.12	91.58	40.24
187	31	50.68	90.60	91.07	39.92
188	28	51.40	91.56	91.98	40.16
189	19	51.20	91.80	92.25	40.60
190	38	51.89	92.83	93.23	40.94
191	7	52.20	92.66	93.19	40.46
192	11	53.75	96.54	97.15	42.79
193	6	53.12	95.85	96.00	42.73
194	6	55.23	99.52	99.90	44.28
195	3	55.70	99.33	99.83	43.63
196	3	54.00	96.23	96.77	42.23
197	2	52.50	94.10	94.70	41.60
198	2	55.50	100.50	100.85	45.00
200	4	55.55	100.08	100.58	44.53
202	1	50.20	88.30	88.60	38.10

N is the number of individuals at the respective stature level. All other measurements in cm.

**Table 4:** Distribution of stature and means of long bone measurements for female (femur, tibia + femur, leg length, tibia length) for each stature level (each cm).

Stature	*n*	Female (n = 2526)			
		Femur	Tibia + femur	Leg length	Tibia
140	2	36.55	65.00	65.05	28.45
142	2	32.15	61.40	61.65	29.25
143	1	37.90	68.10	68.30	30.20
145	2	38.70	69.10	69.35	30.40
146	5	41.12	72.84	72.86	31.72
147	7	40.66	72.23	72.31	31.57
148	7	39.97	71.13	71.26	31.16
149	4	38.33	68.25	68.43	29.93
150	58	39.93	70.95	71.15	31.03
151	10	40.84	72.46	72.44	31.62
152	44	40.47	72.05	72.31	31.58
153	37	40.97	72.98	73.30	32.01
154	33	41.91	74.55	74.82	32.64
155	75	41.06	73.41	73.73	32.36
156	79	41.14	73.56	73.77	32.42
157	61	41.96	74.69	74.96	32.73
158	142	42.29	75.18	75.48	32.89
159	49	43.00	76.26	76.59	33.26
160	272	42.95	76.45	76.79	33.51
161	59	43.79	78.10	78.46	34.32
162	143	43.75	77.88	78.18	34.13
163	159	44.06	78.48	78.83	34.43
164	123	44.46	78.98	79.34	34.52
165	254	44.16	78.81	79.12	34.65
166	73	45.04	80.20	80.54	35.16
167	116	44.96	80.06	80.40	35.10
168	190	45.42	80.90	81.25	35.48
169	45	45.66	81.20	81.55	35.54
170	190	46.21	82.28	82.61	36.07
171	22	46.97	84.00	84.36	37.03
172	69	46.92	83.76	84.06	36.84
173	38	47.36	84.69	85.15	37.33
174	38	47.55	84.64	84.95	37.09
175	46	47.42	84.75	85.14	37.34
176	30	48.00	85.91	86.32	37.91
177	4	49.53	87.95	88.35	38.43
178	17	48.74	87.30	87.65	38.56
179	5	48.96	87.84	88.12	38.88
180	10	50.79	90.49	90.68	39.70
181	3	50.43	90.47	90.67	40.03
187	1	52.50	93.10	93.00	40.60
189	1	49.40	89.20	89.70	39.80

N is the number of individuals at the respective stature level. All other measurements in cm.

**Figure 4:**
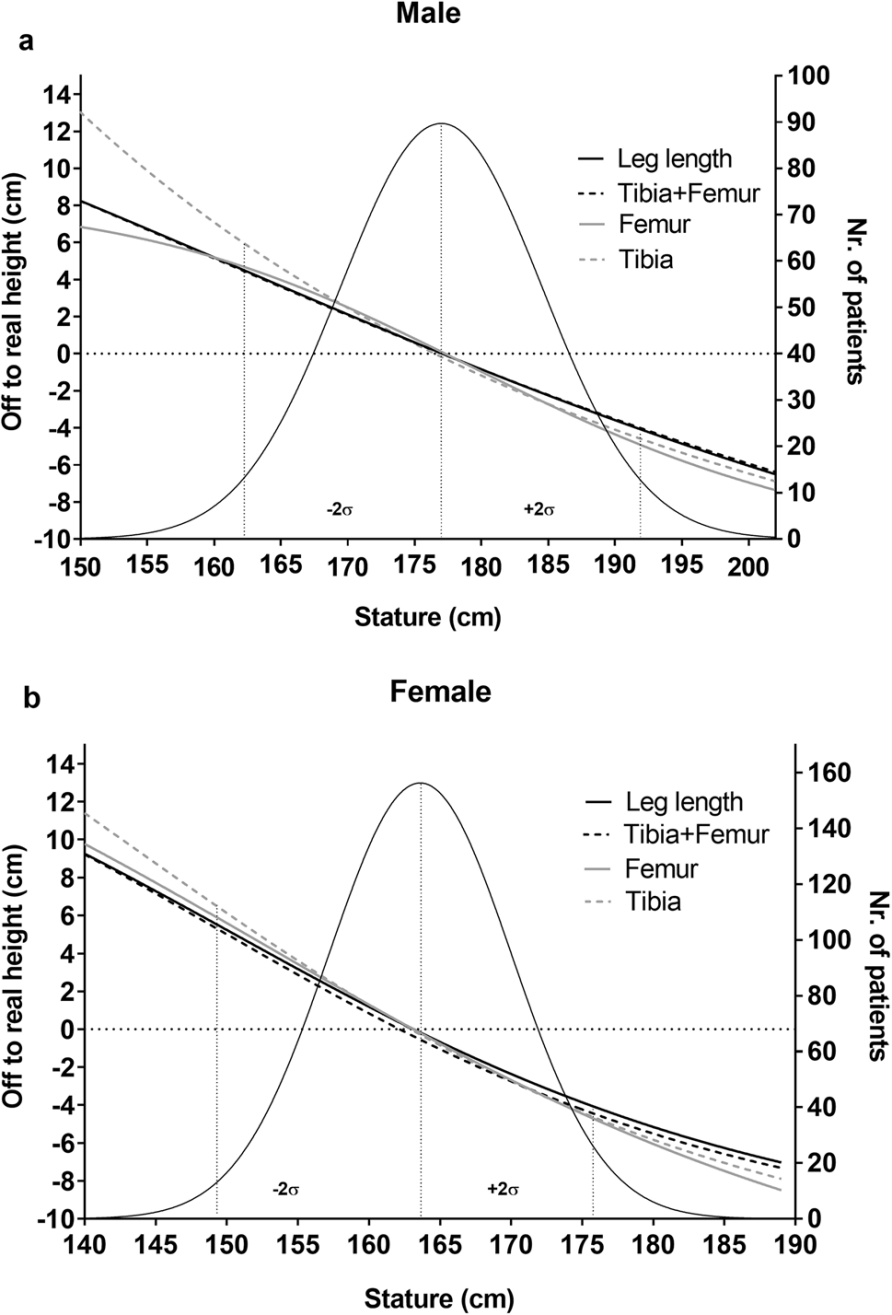
Distribution of stature for males (**a**) and females (**b**) (black Gaussian curve) in our sample. The right vertical axes in (**a**) and (**b**) describe the number of patients with a specific stature value (rounded to cm). The left vertical axes depict the difference between predicted and mean stature value for the four regression formulae derived here (sigmoidal four parameter logistic curve): femur (grey), leg length (black), tibia + femur (black dotted) and tibia (grey dotted). For very short persons (left tail of the distributions), predicted stature was larger than the mean stature and for tall persons (right tail of the distribution) predicted stature was smaller than the mean stature for all four regression formulae. Dotted lines depict the mean as well as ± 2 standard deviations of the stature distributions.

We calculated differences between predicted stature and the mean of the clinically measured stature values for each stature category (each cm) independently, to assess the goodness of fit of the linear regression equations for the different stature categories. We found that the linear equations tended to slightly overestimate stature in short persons, and underestimate stature in tall persons, on average. For example, for male individuals who were two standard deviations (14.8 cm) shorter than the male mean (177 cm), stature was overestimated, on average, by 2.5–3.3% depending on the regression equation (4.7 cm based on the femur equation, 4.4 cm based on the leg length and the tibia + femur equations and 5.9 cm based on the tibia equation). Male individuals who were two standard deviations taller than the male mean were underestimated, on average, by 2.3–2.8% (4.9 cm based on the femur equation, 4.5 cm based on the tibia equation, 4.2 cm based on the leg length equation and 4.0 cm based on the tibia + femur equation, Fig. 4a).

Female individuals who were two standard deviations (13 cm) shorter than the female mean (163 cm) were overestimated, on average, by 3.1–3.7% (5.6 cm based on the femur equation, 5.2 cm based on the leg length equation, 5.1 cm using the tibia + femur equation and 6.1 cm using the tibia equation). Females who were two standard deviations shorter than the female mean were underestimated, on average, by 2.6–3.0% (4.9 cm using the femur equation, 4.8 cm using the tibia equation, 4.2 cm using the leg length equation and 4.6 cm using the tibia + femur equation, Fig. 4b). The mean distribution of stature for males and females is visualized in the Supplementary Material (see Supplementary Fig. S1 online).

## Discussion

In this study, we derive new stature estimation regression formulae based on long bone measurements, which were collected from long leg radiographs of 4200 living Austrians. Measurement was automatized by the software LAMA™^19^, which is an algorithm able to automatically place landmarks utilizing artificial intelligence.

As expected, our findings confirm that different long bone lengths show a high correlation (*r* ≥ 0.76) with stature. Using tibia + femur or leg length resulted in a higher correlation with stature (*r* > 0.84) and hence also in a better predictive capacity of the regression formula compared to formulae using femoral or tibia length alone (*r* > 0.8).

Different stature estimation formulae have been described in the literature for different human populations and geographical areas, such as for Japanese^20^, Thai^21^, Portuguese^5^, Mexicans^22^, White US-Americans^15^ and Native North Americans^23^. The formulae by Trotter and Gleser (1952, 1958) are considered to be most suitable for persons of European ancestry^13,24^, but these formulae were established more than half a century ago. As the secular increase in stature has since led to an absolute increase in average stature in most human populations^25–27^, a review is warranted to assess whether these formulae require adjustment.

Our results show that the regression lines of the present study, which we derived based on a sample of more than 4000 living Austrians, possess a shallower slope and a larger intercept, compared to the formulae derived by Trotter and Gleser (1952, 1958). We suggest that the differences in slopes and intercepts are a consequence of the ongoing secular increase in stature in Europe, where maturation occurs at increasingly younger ages, and absolutely larger adult height is reached. The exploitation of the full growth potential during childhood and adolescence is likely a consequence of reduced poverty, better nutrition and better general health^27^. This phenomenon shifts the population distribution of stature towards higher mean values. At the same time, human bodies, and especially most of our long bones, do not generally grow isometrically^18,28–30^, which implies that the secular increase in stature likely affects the association between stature and the long bones^18,29,31^. In particular, the femur shows positive allometric growth^18^. Consequently, the secular increase in body size could be the reason for the larger intercept and shallower slope in the femoral regression formula derived in this study compared to the estimates by Trotter and Gleser (1952, 1958). An alternative explanation could be that the observed differences in intercepts and slopes are a consequence of genetic differences between samples, or they could be due to non-random sampling in earlier work. Trotter and Gleser (1952, 1958) used samples of military personnel, which might have been truncated, as those too short would not have been accepted into the military. Their female sample (Trotter, Gleser 1952) from the Terry Collection had uncommonly low stature by today ‘s standards.

This study aimed at updating the existing linear regression formulae for stature estimation. Our results indicate that a linear formula is limited in predicting stature accurately for very small and very tall persons. A further limitation of our study is that the exact measurement method and the used anatomical landmarks differ between radiographic measurements as collected here, which is the standard in radiology, and dry bone measurements, as collected in the studies by Trotter and Gleser (1952, 1958) and as usually done in forensics. In the present study, length measurement methods described by *Waldt* et al.^32^ were used as this is the standard in radiological long bone measurements^33,34^. We believe that despite the different measurement methods for long bone length in clinical medicine vs. forensics, these formulae have the potential to be applicable in anthropology and forensics. Dry bone length will likely deviate marginally from bone length measured on radiographs because bones shrink slightly when drying (ca. 2 mm difference in long bone length between fresh and dry bone^15^). In addition, the position of the long bone on an osteometric board will differ marginally from the position of the femur of a person undergoing a radiograph. However, we expect the resulting measurement differences to be small. Future work could estimate the measurement error when assessing long bone length based on dry vs. wet bone vs. radiographs according to the clinical vs. forensic standard for the same person.

To conclude, we found that the regressions derived here have shallower slopes and an increased intercepts compared to formulae from the literature (Trotter and Gleser 1952, 1958). We interpret these differences as a possible consequence of the secular increase in stature. Our study illustrates that AI algorithms are a promising new tool enabling large-scale measurements of bones based on radiographs.

## Methods

The study was approved by the institutional ethics review board (Ethics-Committee of the Vinzenz Group EK: 46/2020) and individual informed consent was waived. All data analysed were collected as part of routine diagnosis and treatment. All experiments were performed in accordance with relevant named guidelines and regulations.

### Study population

Between 2015 and 2020, we performed 17,099 standing antero-posterior LLRs in the Michael Ogon Laboratory for Orthopaedic Research, Orthopaedic Hospital Speising in Vienna, Austria. LLRs and demographic patient information were collected from the institutional arthroplasty registry.

We excluded patients with artificial joints, implants, other kinds of metalwork, posttraumatic or pathologic deformities, metabolic bone diseases, LLRs with no presence or visibility of the calibration ball, patients under 18 years of age, LLRs where the algorithm was unable to identify necessary landmarks and patients where stature was not recorded. In total, 4200 LLRs were measured and included in the final analyses.

### Image acquisition

LLRs were taken as part of the clinical routine, as they are a standard procedure for preoperative planning and for diagnostic purposes. All LLRs were taken on the same device (DigitalDiagnost X-Ray-System 2.1.3, Philips Healthcare Inc., Andover, MA, USA) and each included a 25 mm calibration ball marker, which was placed medially or laterally of the knee joint.

### Automated measurements

Leg-Angle-Measurement-Assistant (LAMA™) software (ImageBiopsy Lab, Vienna, Austria), which automates angle and length measurements on LLRs and annotates the original DICOM images, was used in this study. This program generates numerical outputs for the three linear distance measurements tibial length, femoral length and total leg length. LAMA™ automatically localizes anatomical features of the femur and tibia as well as the calibration ball to assess the landmarks needed to estimate the measurements. The software was designed to suppress the output if landmarks cannot be placed appropriately. Length calibration was performed by segmenting the calibration ball and calculating a magnification factor based on the size of the calibration ball and the diameter of the segmentation (pixel units).

**Figure 5:**
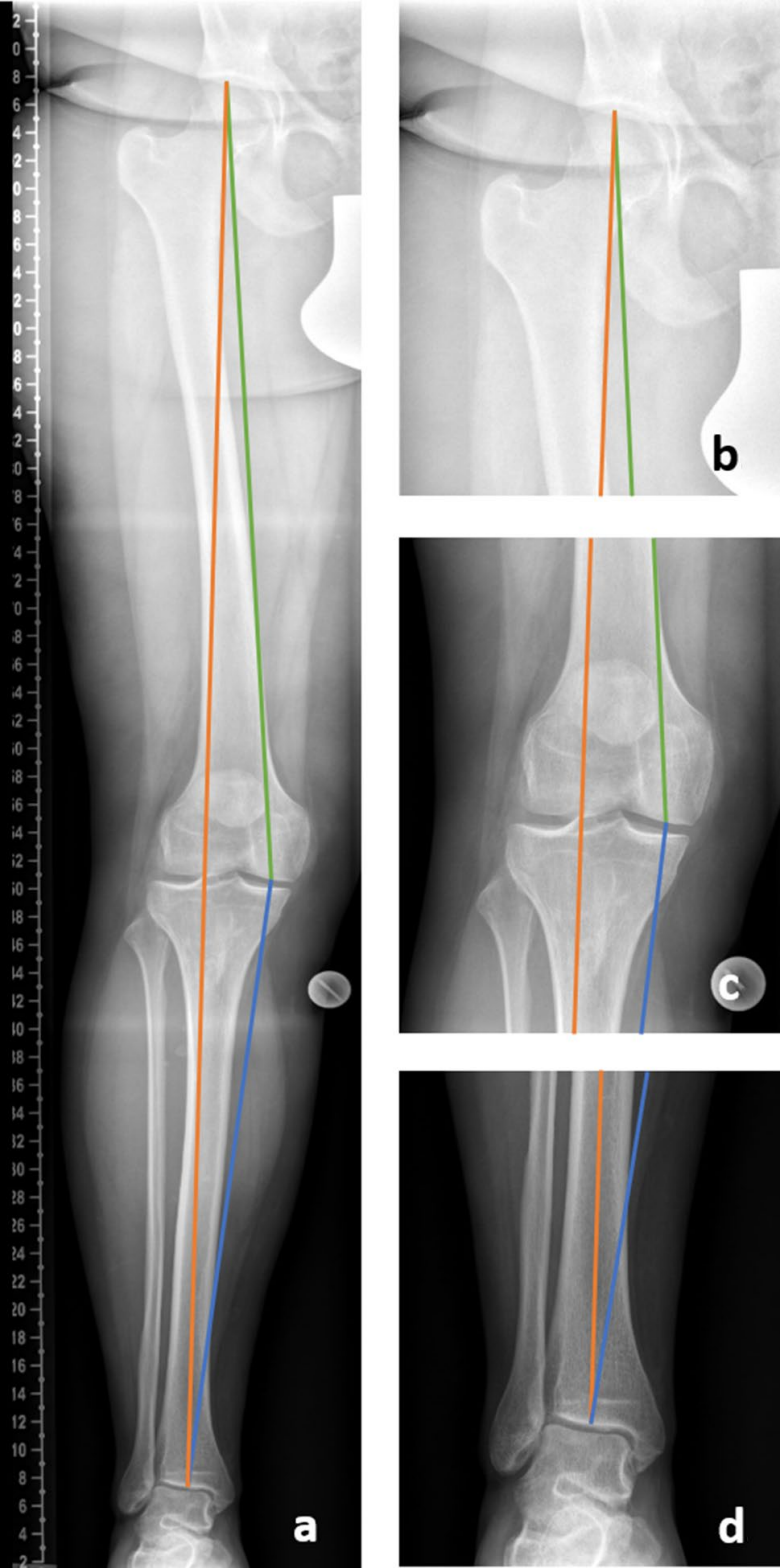
Biometric linear distance measurements taken on the long leg radiographs. Leg length (orange line): distance between the top of the femoral head and the midpoint of the tibial plafond; maximum femoral length (green line): distance between the top of the femoral head and the distal portion of the medial femoral condyle; tibial length (blue line): distance between the distal portion of the medial femoral condyle and the midpoint of the tibial plafond.

For all LLRs the following linear distance measurements were computed (Fig. 5)^32^. Leg length (measured as linear distance between top of the femoral head and midpoint of the tibial plafond), maximum femoral length (top of the femoral head–bottom of the femoral medial condyle), and tibial length (midpoint of proximal tibial joint line–midpoint of the tibial plafond).

### Validation

The AI algorithm applied in this study was validated in a previous study on a smaller dataset of 289 LLRs and showed excellent intra-class-correlation between manually measured and automated measured lengths^19^.

### Comparison to existing formulae

The formulae derived in the present study were subsequently compared to existing formulae published by Trotter and Gleser in the 1950s (Trotter and Gleser 1952, 1958). Trotter and Gleser measured samples of US military personnel from the Korean War and from World War II. Stature measurements were recorded at the time of induction into military service. Long bone measurements were conducted before final burial. Formulae for females were derived by Trotter and Gleser (1952)^15^ based on corrected equations from the Terry Collection samples (Smithsonian Institution, Washington D.C.).

### Statistical analysis

Four ordinary least squares linear regression equations were estimated for stature as dependent variable and for femur, tibia, femur + tibia and total leg length, respectively, as predictor variable. Regressions were estimated separately for males and females. Correlation coefficients between stature and the three variables, leg length, femur length, and tibia length, respectively, were calculated, separately for males and females.

To assess the goodness of fit of the linear regression equations, we computed differences between the predicted value and the mean of the clinically estimated stature values for each stature category (for each cm). The resulting differences capture how well the linear model approximates the mean of the clinical measurements for each stature category. To plot the resulting differences, they were approximated by a logistic sigmoidal function (4 parameter logistic regression).

*P *values < 0.05 were considered statistically significant throughout the study. All analyses were performed using IBM-SPSS® version 25 and GraphPad Prism® version 8.

## Supplementary Information

Supplementary Information.

## Data Availability

The data that support the findings of this study are available on request from the corresponding author. The data are not publicly available due to privacy or ethical restrictions.
