# Supplementary material for: Body height estimation from automated length measurements on standing long leg radiographs using artificial intelligence
Source: Sci Rep. 2023 May 25;13:8504. doi: 10.1038/s41598-023-34670-2 (PMC10213042; doi:10.1038/s41598-023-34670-2)
Supplement: Supplementary file 1 — Supplementary Information. [file 41598_2023_34670_MOESM1_ESM.docx]

**Body height estimation from automated length measurements on standing long leg radiographs using artificial intelligence**

Sebastian Simon, MD^1,2^, Barbara Fischer, PhD^3^, Alexandra Rinner^1^, Allan Hummer, PhD^4^, Bernhard JH. Frank, MD^1^, Jennyfer A. Mitterer, MD^1^, Stephanie Huber, MD^1,5^, Alexander Aichmair, MD^1,2^, Gilbert M. Schwarz, MD ^1,5^, Jochen G. Hofstaetter, MD^1,2^ *

^1^ *Michael Ogon Laboratory for Orthopaedic Research, Orthopaedic Hospital Vienna-Speising, Speisinger Straße 109, 1130 Vienna, Austria*

*^2^ 2^nd^ Department, Orthopaedic Hospital Vienna-Speising, Speisinger Straße 109, 1130 Vienna, Austria*

*^3^ University of Vienna, Department of Evolutionary Biology,* *Unit for Theoretical Biology, Djerassiplatz 1, 1030 Vienna, Austria*

*^4^ ImageBiopsy Lab GmbH, Zehetnergasse 6/2/2, 1140 Vienna, Austria*

*^5^ Medical university of Vienna, Center for Anatomy and cell Biology, Währingerstraße 13, 1090 Vienna, Austria*

**Supplementary information**

Supplementary figure S1

**
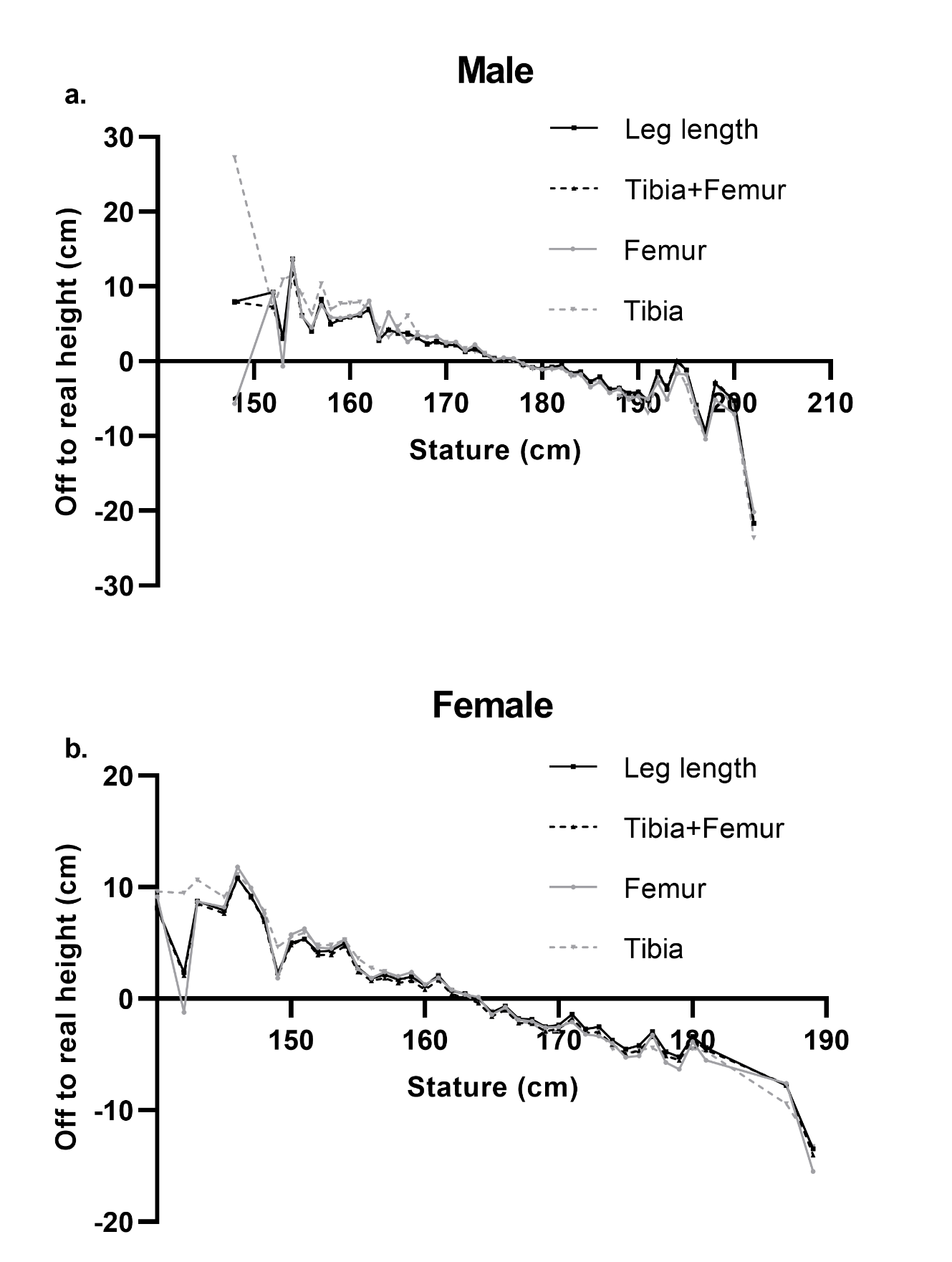
Mean difference to real height**

**Figure legend:**

Suppl. Fig. S1. Differences between mean stature and predicted stature, based on our regression equations, for males (a.) and females (b.) in our sample. The vertical axes depict the difference between predicted and actual mean stature value for the four regression formulae derived here (means per stature category, i.e. each cm). For very short persons, predicted stature was larger than the actual stature and for tall persons predicted stature was smaller than the actual stature values for all four regression formulae.
